# Rapid purification and multiparametric characterization of circulating small extracellular vesicles utilizing a label-free lab-on-a-chip device

**DOI:** 10.1038/s41598-023-45409-4

**Published:** 2023-10-25

**Authors:** Manju Sharma, Maulee Sheth, Holly M. Poling, Damaris Kuhnell, Scott M. Langevin, Leyla Esfandiari

**Affiliations:** 1 Department of Biomedical Engineering, College of Engineering and Applied Sciences, University of Cincinnati, Cincinnati, OH USA; 2 Division of Pediatric General and Thoracic Surgery, Cincinnati Children’s Hospital Medical Center, Cincinnati, OH USA; 3 Department of Environmental and Health Sciences, College of Medicine, University of Cincinnati, Cincinnati, OH USA; 4 Larner College of Medicine, University of Vermont, Burlington, VT USA; 5 University of Vermont Cancer Center, Burlington, VT USA; 6 Department of Electrical Engineering and Computer Science, College of Engineering and Applied Sciences, University of Cincinnati, Cincinnati, OH USA; 7 University of Cincinnati Cancer Center, Cincinnati, OH USA

**Keywords:** Biomarkers, Nanoscience and technology, Lab-on-a-chip, Non-coding RNAs, Biomedical engineering

## Abstract

Nano-scale extracellular vesicles are lipid-bilayer delimited particles that are naturally secreted by all cells and have emerged as valuable biomarkers for a wide range of diseases. Efficient isolation of small extracellular vesicles while maintaining yield and purity is crucial to harvest their potential in diagnostic, prognostic, and therapeutic applications. Most conventional methods of isolation suffer from significant shortcomings, including low purity or yield, long duration, need for large sample volumes, specialized equipment, trained personnel, and high costs. To address some of these challenges, our group has reported a novel insulator-based dielectrophoretic device for rapid isolation of small extracellular vesicles from biofluids and cell culture media based on their size and dielectric properties. In this study, we report a comprehensive characterization of small extracellular vesicles isolated from cancer-patients’ biofluids at a twofold enrichment using the device. The three-fold characterization that was performed using conventional flow cytometry, advanced imaging flow cytometry, and microRNA sequencing indicated high yield and purity of the isolated small extracellular vesicles. The device thus offers an efficient platform for rapid isolation while maintaining biomolecular integrity.

## Introduction

Small extracellular vesicles (sEVs) are membrane-encapsulated biological vessels ranging from 40–150 nm in diameter. sEVs are released by cells into the extracellular space and can be found in essentially all biofluids including blood^1,2^, urine^3^, saliva^4^, human semen^5^, breast milk^6^, and cerebrospinal fluids^7^. These nanoscale vesicles are capable of horizontal transfer of their biomolecular cargo, e.g. microRNA (miRNA), and can thus function as intercellular signaling vectors^8^. Compared to other biomarkers, sEVs provide a high degree of sensitivity and specificity owing to their excellent stability^9^. The unique properties of sEVs, including their enrichment of RNA cargo through an active loading process, abundance in circulation, reproducible detection, and the ability to reflect their parental cells’ states, make them a unique set of circulating biomarkers for early diagnosis in liquid biopsy for diseases such as cancers, neurodegenerative, infectious, and metabolic diseases^9–12^. sEVs isolation has, however, been a challenging task due to their nanoscale size, high heterogeneity, and physicochemical properties^13^.

Each isolation technique exploits a particular trait (or traits) of sEVs, e.g. their density, shape, size, and/or surface proteins^11^. However, most current sEVs isolation techniques, including combination approaches, suffer from limited yield, low throughput, issues with purity, and/or lengthy isolation times^14–19^. Microfluidic devices could potentially overcome many of the aforedescribed challenges by providing significant reductions in sample volume requirement, isolation time, and reagent consumption^20^. Microfluidic devices target physical properties at the microscale level and enable rapid and high throughput isolation of sEVs by employing external forces. Electrokinetic based microfluidics are one set of such devices that isolate sEVs based on external electrical forces without the need to tag or label molecules of interest^21,22^. The dielectrophoresis (DEP) phenomenon can achieve specific isolation based on the dielectric properties and size distribution of sEVs under a non-uniform electric field, in a rapid and label-free manner. This non-uniform electric field can be generated by the application of an alternating current across an array of electrodes^23,24^ or by using an insulator-based dielectrophoretic (iDEP) approach using obstacles e.g. micro-pillars in microfluidic channels^25^. The iDEP approach can manipulate biomolecules by preserving device functionality despite the fouling effect at the surface of electrodes^26^. However, majority of these devices require high operational voltage (~ 100 V/cm), which could potentially denature molecules of interest^27^. Additionally, while these devices have shown promising attributes, the high cost of fabrication, need for sample dilution, and susceptibility of micro- and nano-scale channels to clogging are some inherent challenges associated with them^17^.

**Figure 1:**
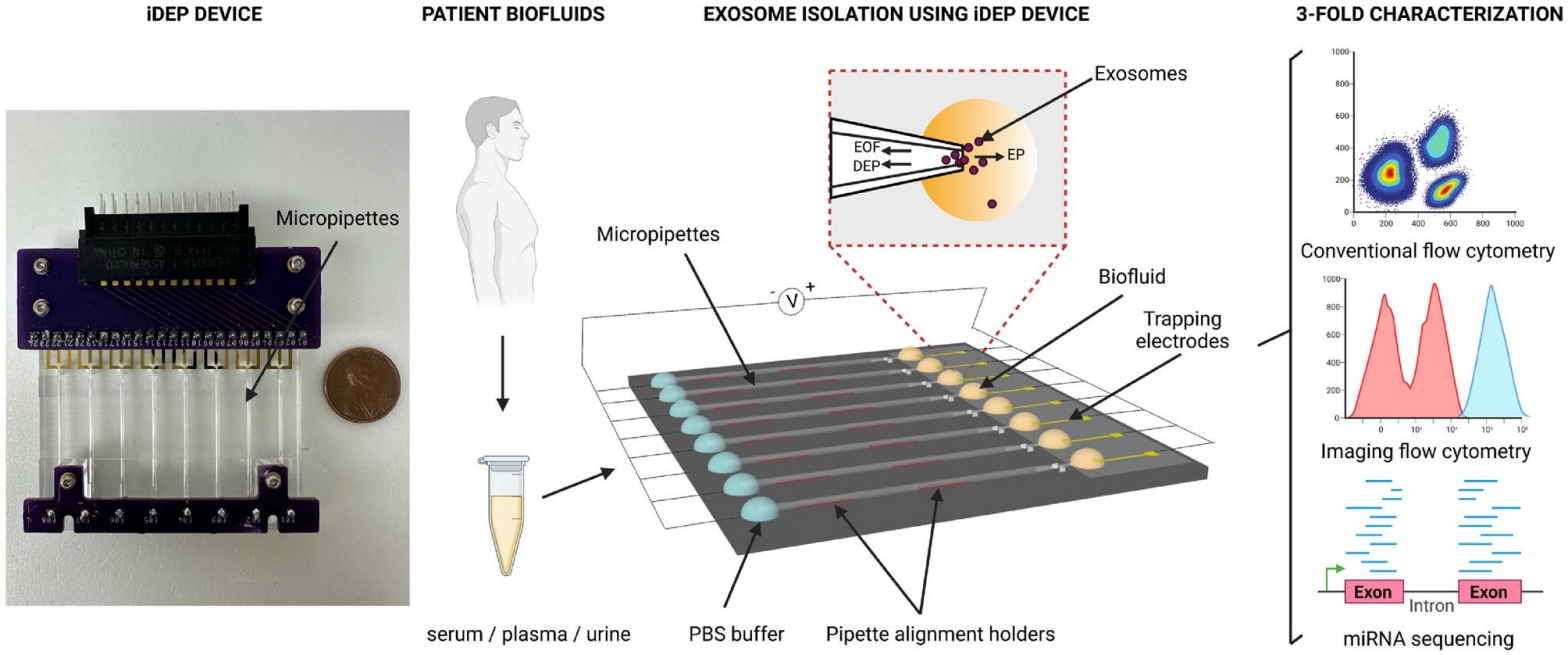
Device layout and graphical outline of experimental flow. Small extracellular vesicles (sEVs) were simultaneously extracted from human biofluids using the insulator-based dielectrophoretic (iDEP) device, wherein a trapping zone was created at the tip region of micropipettes by balancing the dielectrophoretic (DEP) force with two electrokinetic forces, including electroosmosis (EOF) and electrophoresis (EP). sEVs isolates were comprehensively characterized using flow cytometry, ImageStream^x^, and microRNA (miRNA) sequencing.

Our group has developed a new class of iDEP device for rapid and selective entrapment of nanoparticles based on their size and unique dielectric properties. The device, consisting of an array of micropipettes, is capable of isolating nanoparticles from small sample volumes by applying a significantly low electric field (~ 10 V/cm) across the length of the pipettes^28^. The miniscule conical pore geometry of the micropipettes enables isolation of sEVs without the need for sample dilution, hence allowing for downstream analyses with uncompromised yield. We previously demonstrated the capability of our iDEP device to isolate sEVs from small sample volumes (~ 200 μL) of conditioned cell culture media and biofluids from healthy donors within 20 min^29^. Here we comprehensively assessed the yield and purity of isolated sEVs using the next generation of the iDEP device from biofluids of cancer patients, including serum, plasma, and urine, using multiparametric characterization via flow cytometry and next generation miRNA-sequencing (miRNA-seq). Figure 1. Illustrates the schematic and principal operation of the device along with the sEVs characterization workflow. MISEV 2018 and MIFlowCyt-EV guidelines were followed throughout the study for sEVs isolation and relevant characterization^30,31^.

## Results

### sEVs isolation utilizing the iDEP device

sEVs from serum, plasma, and urine samples were purified in PBS using the iDEP device and characterized by performing nanoparticle tracking analysis (NTA). The median concentration for serum was 1.22 × 10^11^ particles/mL with a range from 6.2 × 10^10^ particles/mL to 5.46 × 10^11^ particles/mL. Median sEVs concentration was 1.18 × 10^10^ particles/mL (range: 3.45 × 10^9^ particles/mL to 7.73 × 10^10^ particles/mL) and 5.93 × 10^11^ particles/mL (range: 9.55 × 10^10^ to 1.09 × 10^12^) for urine and plasma, respectively (Supplemental Tables S1 and S2). NTA results of sEVs isolated using the iDEP device were also found to be comparable to previous studies^32,33^. Transmission electron microscopy (TEM) was used to further confirm the presence of sEVs (Supplemental Fig. S1). Distribution of tetraspanins across sEVs within a sample was assessed by performing 3-color dSTORM super resolution microscopy against CD63, CD9, and CD81 markers (Supplemental Fig. S2). With 59.5% sEVs found to be triple positive for all three markers, tetraspanin distribution was found to be comparable to positive control A549 NSCLC derived sEVs from ATCC Inc. with 47.3% triple positive sEVs (Supplemental Fig. S2).

### Multiparametric analysis of purified circulating sEVs by flow cytometry

#### Conventional flow cytometry

**Figure 2:**
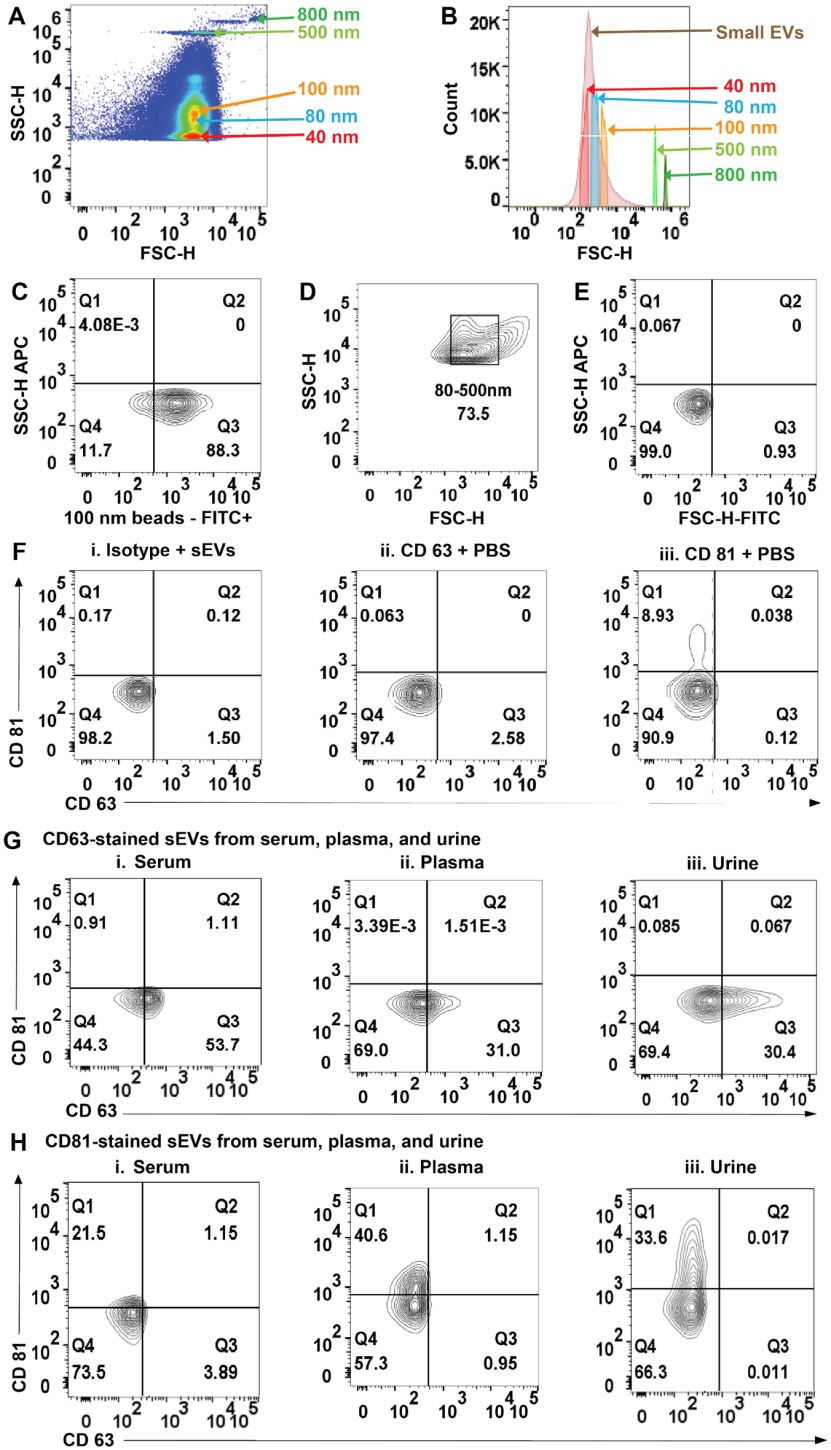
(**A**) FSC-H versus SSC-H of PS-COOH beads analysis on high-resolution flow cytometry represents the resolution of beads size in PS-COOH mix. (**B**) Histogram illustrating different sizes of PS-COOH beads and serum-derived small extracellular vesicles (sEVs). (**C**) Representative plots of PS-COOH-100 nm beads fluorescence-FITC. (**D**) Representative FSC-H versus SSC-H of sEVs isolates from human plasma. (**E**) Representative FSC-H versus SSC-H of unstained sEVs demonstrating gating. (**F**) Representative plots as negative controls i. Isotype-stained sEVs. ii. CD63 antibody in PBS and iii. CD81 antibody in PBS. (**G**) Representative dot plots of sEVs stained for CD63 from i. serum ii. plasma and iii. urine. (**H**) Representative plots of sEVs stained for CD81 from i. serum ii. plasma and iii. urine.

iDEP sEVs isolates from serum, plasma, and urine of cancer patients were characterized using flow cytometry. The initial gate was acquired by determining the resolution of 40–800 nm size range in a PS-COOH beads mix (Fig. 2A). Serum-derived sEVs were overlaid to obtain a comparative histogram illustrating variation in size of beads and sEVs (Fig. 2B). 100 nm-stained PS-COOH beads were acquired to aid in proper gating for positive signals (Fig. 2C). The data thus obtained was used to create an 80–500 nm gate encompassing the sEVs to be characterized (Fig. 2D). Once the sEVs gate was created, unstained samples were used to gate out the negative signals (Fig. 2E). Negative controls of isotype-stained sEVs were acquired to verify non-specific binding, and antibodies CD63 and CD81 in PBS were acquired to verify no interference from free floating antibodies with sample acquisition (Fig. 2F).

sEVs isolates were analyzed for sEVs-associated tetraspanins CD63 and CD81 using dot plots, with the percentages yielding quantization for each marker. 55% of isolated sEVs from serum, 31% from plasma, and 30% from urine were observed to be positive for CD63 (Fig. 2G). Similar analysis showed 22% sEVs from serum, 41% from plasma, and 34% from urine to be positive for CD81 (Fig. 2H). Conventional flow cytometry (cFCM) analysis has previously indicated 38.1% sEVs to be positive for both CD63 and CD81 when using differential ultracentrifugation (DU) for isolation from Broncho Alveolar Lavage (BAL) fluid samples^34^. cFCM results using the iDEP device for the same markers were found to be in a comparable range (Fig. 2G,H).

#### Imaging flow cytometry using ImageStream^x^

**Figure 3:**
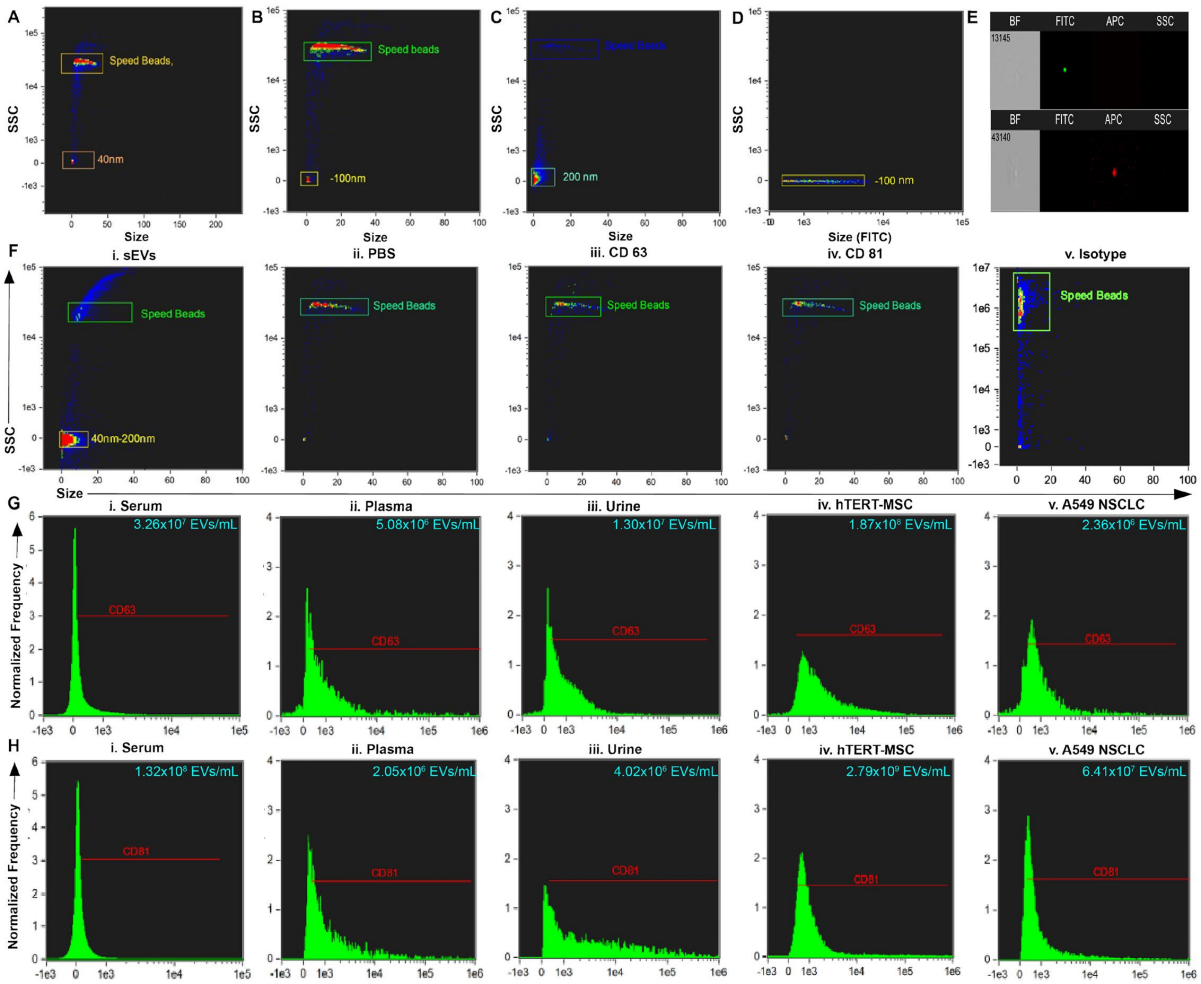
(**A**–**C**) Size versus SSC of beads and calibration speed beads. (**D**) Size versus FITC illustrating the location on side scatter that is positive for fluorescent beads. (**E**) Representative dot plot of FITC vs. Scatter Intensity (top) and APC vs. Scatter Intensity (bottom). (**F**) Representative size vs. SSC plot of i. small extracellular vesicles (sEVs) purified from biofluid and negative controls: ii. PBS iii. antibody CD63 iv. antibody CD81 and v. Isotype. (**G**) Representative histogram of CD63+ sEVs from i. serum, ii. plasma, iii. urine, and positive controls: iv. hTERT-immortalized mesenchymal stem cell (MSC) sEVs, and v. A549 non-small cell lung carcinoma (NSCLC)-derived sEVs. (**H**) Representative histogram of CD81+ sEVs from i. serum, ii. plasma, iii. urine, and positive controls iv. hTERT-immortalized MSC sEVs, and v. A549 NSCLC sEVs.

To further demonstrate the capacity and utility of the iDEP device, sEVs isolates were characterized using ImageStream^x^. Figure 3A–D represents the dot plots of side scatter versus size of unstained and stained calibration beads used to determine the region of sEVs presence. The acquisition region thus created was tested using sEVs extracted from biofluids and negative controls, including PBS, antibody CD63, antibody CD81 and Isotype (Fig. 3F). sEVs lysis controls are reported in Supplemental Fig. S3. Figure 3E represents the single-event visual interrogation for each marker used to confirm the gating of populations via along with the assessment of the scatter characteristics of labeled particles of known size.

Analysis of the scatter profiles of samples revealed high CD63+ expressions with 3.26 × 10^7^ EVs/mL isolated from serum, 5.08 × 10^6^ EVs/mL from plasma, and 1.3 × 10^7^ EVs/mL from urine as seen from the representative histograms (Fig. 3G: i–iii). CD63+ expressions for EVs isolated using the iDEP device were found to be comparable to CD63+ expression from purified hTERT-immortalized mesenchymal stem cells (1.87 × 10^8^ EVs/mL) and A549 NSCLC (2.36 × 10^6^ EVs/mL) sEVs purchased from ATCC Inc as positive controls (Fig. 3G: iv, v).

Similar analysis for CD81+ objects also revealed high yield with 1.32 × 10^8^ EVs/mL isolated from serum, 2.05 × 10^6^ EVs/mL from plasma, and 4.02 × 10^6^ EVs/mL from urine (Fig. 3H: i–iii). Isolation using the device was again found to be comparable to the positive controls including hTERT-immortalized mesenchymal cells (2.79 × 10^9^ EVs/mL) and A549 NSCLC cells (6.41 × 10^7^ EVs/mL) sEVs obtained from ATCC Inc. (Fig. 3H: iv, v). Imaging flow cytometry (iFCM) analysis for iDEP device isolation was also found comparable to sEVs isolated using three commonly used techniques—DU, size exclusion chromatography (SEC), and polymer-based precipitation (PBP). For instance, iFCM analysis for isolation using the iDEP device revealed 5.08 × 10^6^ of CD63+ EVs/mL from plasma samples, which was comparable to CD63+ EV expressions that have been previously reported for isolation when using DU (~ 1 × 10^7^ EVs/mL), SEC (3 × 10^7^ EVs/mL), and PBP (~ 2.2 × 10^7^ EVs/mL) from plasma of liver transplant subjects^35^. Population statistics (Fig. 3H: i–iii) were also found to be comparable to a previous study on iFCM analysis of sEVs isolated using DU from mesenchymal stem cell derived culture media which reported 1.5 × 10^6^ CD81+ EVs/mL^36^.

### microRNA sequencing

As an additional form of iDEP device validation, miRNA was extracted from the isolated sEVs obtained from three distinct biofluids and a variety of cancer types and sequenced. The mean total RNA concentration obtained from sEVs isolated using the iDEP device was 96 ng/mL for serum, 99 ng/mL for plasma, and 105 ng/mL for urine samples (RNA quality control results are provided in Supplemental Tables S1 and S4). These were found to be comparable to the mean exosomal miRNA concentrations from previous studies. For instance, RNA concentration for plasma sEVs of lung cancer patients has been reported to be 158.6 ng/mL when using a 2-step procedure combining SEC and magnetic activated cell sorting^32^. Another study reported a 165 ng/mL RNA concentration for sEVs from human cell-free serum samples isolated using DU^33^. Human urinary sEVs have been reported to have 83 ng/mL and 55 ng/mL RNA concentrations for isolation using DU and a DU-SEC combination, respectively^37^.

**Figure 4:**
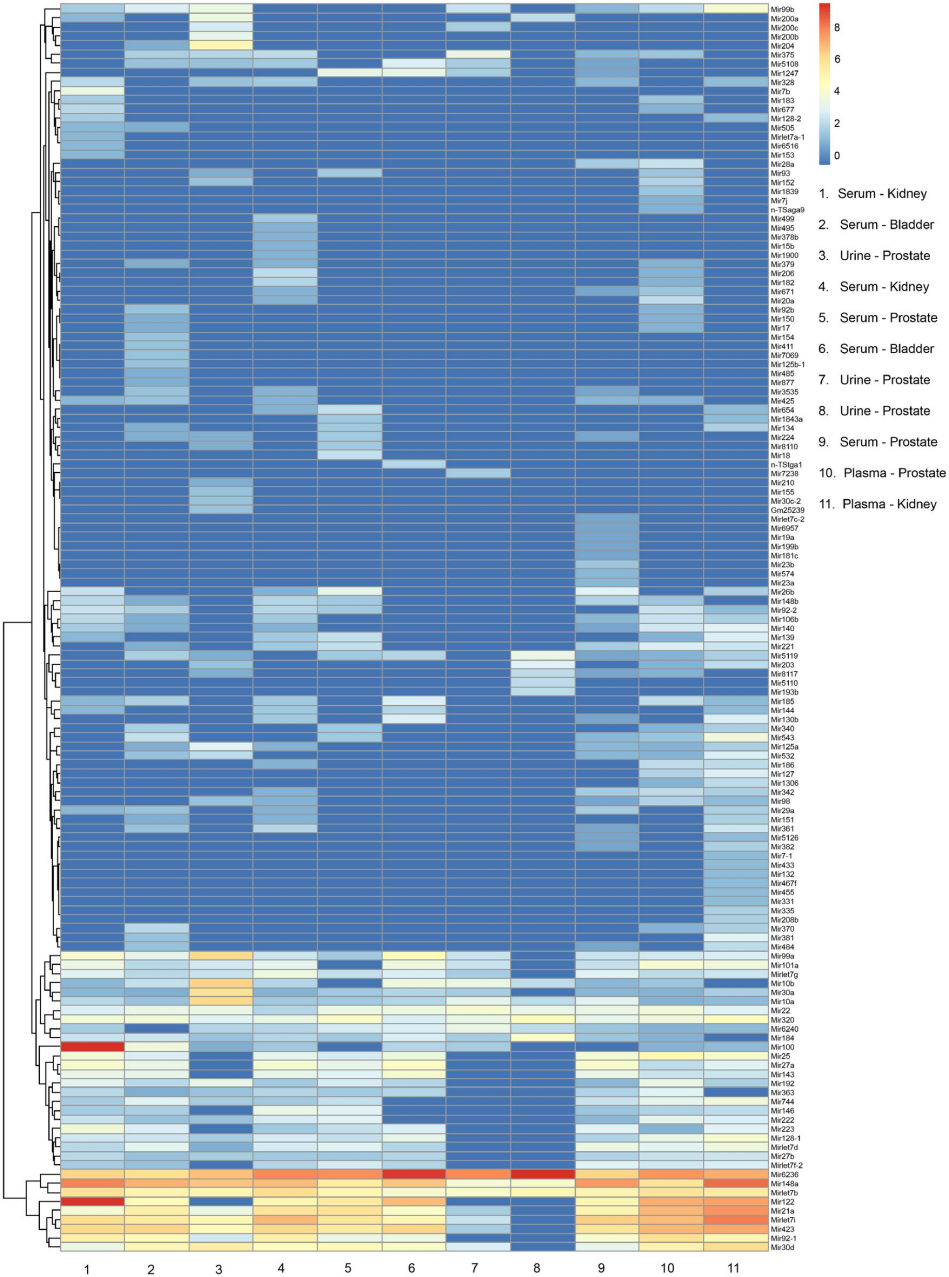
Heatmap of microRNA transcripts isolated from serum, plasma and urine small extracellular vesicles (sEVs) from patients with genitourinary tract cancers. Columns represent individual samples. Legend shows type of biofluid and cancer site for each sample. Rows represent microRNA gene transcripts. Color bar scale represents miRNA enrichment. RStudio Desktop (version 2023.06.2 + 561, accessible at https://posit.co/download/rstudio-desktop/) was used for heatmap generation.

Following miRNA-seq, 137 distinct mature miRNA transcripts were uniquely mapped to the human genome (hg38) across samples, thus validating the device for inclusion in miRNA biomarker analysis workflows^38,39^. A heatmap of the miRNA transcripts highlights the expression profiles between the sEVs miRNA cargo across samples (Fig. 4). In this feasibility dataset, the most highly enriched miRNAs were *hsa-miR-6236*, which has had little characterization; *hsa-miR-148a*, a known tumor suppressor; and *hsa-let7b* which has also been implicated in several cancers including lung, liver, breast and ovarian^40–46^. The clustering dendrogram for the columns is provided as Supplemental Fig. S4. sEVs collected from urine samples branched distinctly, while those from serum and plasma were mixed, likely due to their common blood origins.

**Figure 5:**
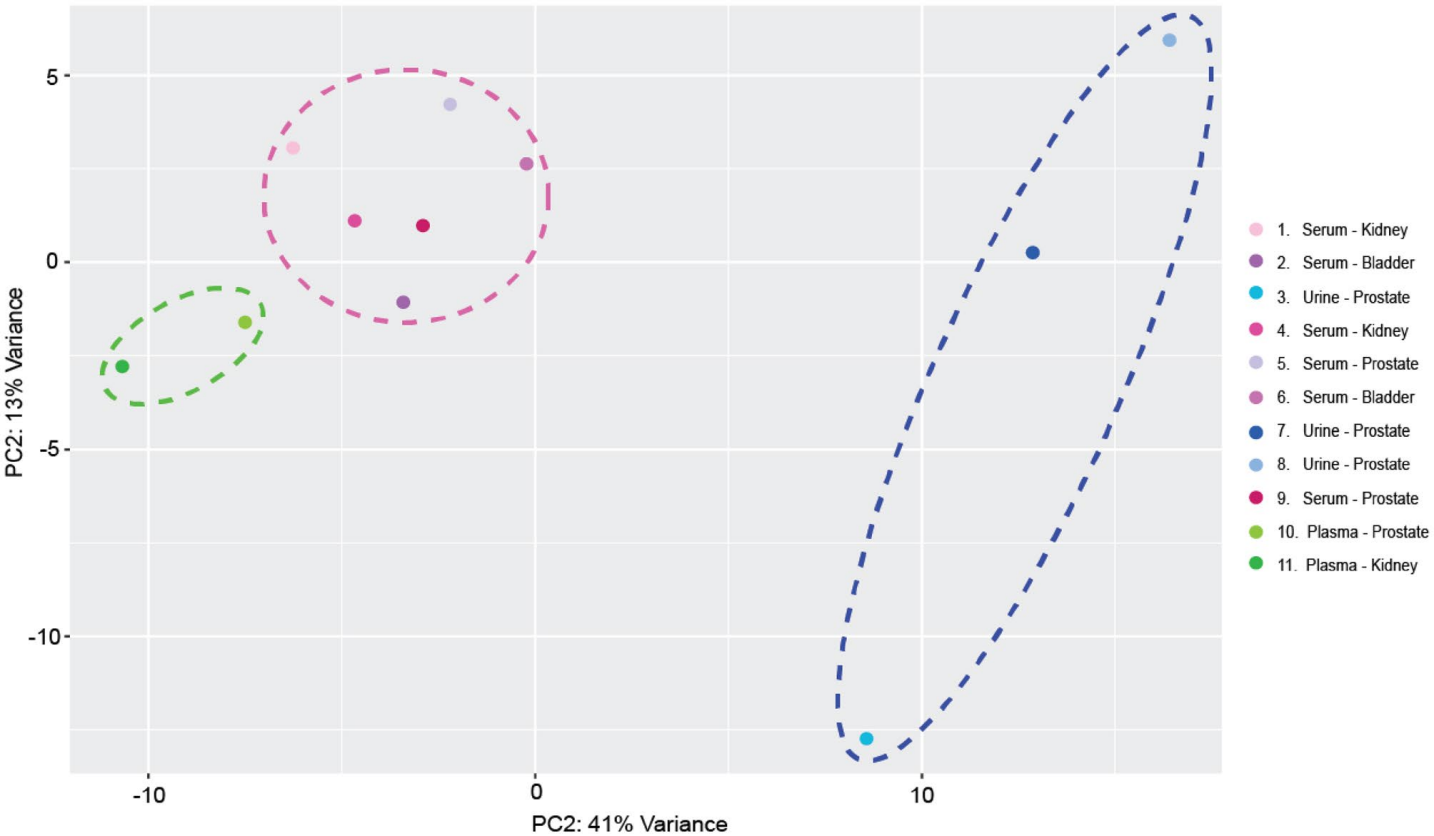
Scaled centered principal component analysis of exosomal miRNA profiles. Samples clustered based on exosomal origin. Green dashed oval highlights samples extracted from plasma. Pink dashed circle highlights samples extracted from serum. Dark blue dashed oval highlights samples extracted from urine.

In order to visualize the likeness between transcriptomic profiles of the samples, principle component analysis was also performed. With 54% variance in 2D, sEVs collected from urine segregated to +PC1, while samples collected from serum and plasma intermixed in a −PC1 cluster (Fig. 5). Furthermore, we observed plasma-based samples clustering on −PC2, while most coming from serum remained on +PC2 (Fig. 5). Interestingly, as demonstrated with the clustering dendrogram, samples again tended to group based on biofluid rather than the type of cancer. Importantly, the transcriptomic analysis demonstrated that iDEP sEVs isolation is compatible with downstream characterization and analysis of human sEVs miRNA cargo and potential use in clinical biomarker analysis.

## Discussion and conclusions

In this study, we have demonstrated the capacity and efficiency of a low-voltage, label-free iDEP device in isolating sEVs from serum, plasma, and urine from cancer patients by performing sub-micron particle detection and multiparametric characterization using cFCM and advanced iFCM. Analysis of dot plots from cFCM and expression profiles from iFCM allowed us to quantify the percentage and objects/mL, respectively, of purified sEVs positive for EVs-specific markers CD63 and CD81 in each sample. Previous studies have shown that owing to its lower size cut-off (270–300 nm), cFCM can detect less than 1% of bulk EVs as compared to the gold-standard quantitative immune-TEM^47,48^. High-resolution flow cytometry on the other hand, resulted in only a 15-fold underestimation of EVs concentration compared to TEM in a biological sample, owing to its improved 150–190 nm lower size limit. This resulted in an overall 20-fold difference between conventional and high-resolution cytometry^48^. In the present study, we employed both cFCM and high resolution iFCM to validate sEVs isolation using the iDEP device. cFCM, which has been widely used to characterize sEVs, enabled us to compare the yield with other conventional isolation techniques reported previously^34^. Additionally, iFCM, with an improved limit of detection of 70 nm and advantages including time delay integration of each particle, low noise charge-coupled device cameras, and detection of even dimly fluorescent particles due to triggering in all channels, allowed for characterization of sEVs with improved sensitivity^49^. Together, both flow techniques revealed high yield and purity of sEVs from all three biofluids and were found to be comparable to previous flow cytometric studies using conventional sEVs isolation methods^34–36^.

When comparing NTA and flow cytometry results, concentration of sEVs detected with NTA exceeded CD63+ and CD81+ imaging flow cytometric events by anywhere between one to six orders of magnitude, depending on the sample, source, and target marker. This could be indicative of the potential presence of untagged EVs or isolated non-sEVs particles within the same size range. van der Pol et al*.* have also previously found 15 times lower EV counts with flow cytometry as compared to NTA due to different limit of detections between the two techniques and the potential detection of non-EV structures such as lipoproteins or protein aggregates by NTA^50^. Additionally, it has now been established that human biofluids contain multiple subpopulations of EVs that carry different tetraspanins as opposed to initial work that suggested universal enrichment of these membrane proteins in EVs isolated across cell types^51^. This was further supported by tetraspanin co-localization analysis obtained from 3-color dSTORM super resolution imaging performed on cancer serum sEVs isolated using the iDEP device. Such heterogenous tetraspanin distribution obtained using microscopy have also been reported previously^52–56^. For more accurate comparisons, follow up flow cytometric characterization studies simultaneously targeting multiple EV tetraspanins such as CD63, CD81, and CD9 are required. Additionally, while NTA results indicated a mean size range of ~ 100 nm across samples, TEM quantification showed mean particle diameters of ~ 30 nm. This is in agreement with a study by Mizenko et al*.* where TEM images indicated a mode diameter of ~ 35 nm as compared to an apparent mode between 100 and 150 nm from NTA for SK-OV-3 human OvCa cell-line EVs^57^. This non-agreement in size distributions between the two techniques can hence be attributed to the fact that NTA is capable of only detecting EVs larger than 70 nm in nominal diameter owing to the low scattering of organic particles while TEM has been found to show a logarithmic-linear particle count vs. size down to ~ 30 nm^48,57^. Interestingly, the size range obtained from TEM is also indicative of the potential presence of exomeres, a new class of EVs that lack the lipid bilayer and are significantly smaller (< 50 nm). Currently, exomeres are primarily isolated by asymmetric-flow field-flow fractionation (AF4), ultracentrifugation, or combination approaches^58^. Additional studies utilizing the iDEP device are needed to characterize and distinguish the exomeres from sEVs. The device thus has potential for isolation of heterogenous groups of the smallest EVs, down to 30 nm, with more selective entrapment of the 30 to 150 nm size sEVs range.

For RNA quality control, it is worthwhile to note that while RNA concentrations were found comparable to previous studies, the sample volume utilized for the iDEP isolation was substantially lower in certain cases. For instance, while urine exosomal RNA concentration from iDEP isolation was found similar to isolation using a DU-SEC combination strategy, the iDEP platform with its low sample volume requirement, only utilized 1 mL sample urine as opposed to 50 mL for the combination approach^37^. Furthermore, through next generation RNA sequencing, human sEVs miRNA cargo was successfully sequenced and aligned, demonstrating the compatibility of downstream transcriptomic analysis with the iDEP platform. While the primary aim of RNA sequencing was to confirm the presence of human genes in sEV isolates, several miRNAs including *hsa-miR-6236*, *hsa-miR-148a*, and *hsa-let7b,* that are differentially secreted in cancers were found to be detectable at substantially high levels across samples, highlighting the utility of our platform for miRNA-based applications^40–46^. Additionally, sample origin-based PCA clustering indicated the compatibility and efficiency of our device with different types of biofluids for downstream studies involving miRNAs in sEVs associated with cancers as diagnostic and prognostic markers. Overall, this affirms our isolation methodology as a viable alternative to those currently established.

The comprehensive characterization and quantification of vesicles along with the use of TEM and NTA performed in this study verified that sEVs were successfully isolated from biofluids while preserving sEVs integrity. This characterization provided further validation of the quality and integrity of sEVs isolated by the iDEP platform to our previous study where the presence of sEVs in health donor serum and cell culture media was confirmed by Western blot analysis against sEV-specific cytosolic protein marker TSG101 and membrane protein marker CD81^29^. While yield and purity of sEVs isolated using the iDEP device were found to be comparable to techniques including DU and SEC, the platform overcomes some of the key limitations in EV isolation by having added advantages including shorter isolation time (~ 20 min) and need for no specialized equipment, large sample volumes (200 μL), or sample dilution. The device is capable of isolating bulk sEVs in an efficient and unbiased label-free manner, enabling EV-based biomarker applications and cell-free therapies in a clinical setting. The iDEP device thus has strong potential as a liquid biopsy platform for isolation of sEVs to contribute to the development of EV-based diagnostic and monitoring tools.

## Materials and methods

All chemicals were purchased from Sigma-Aldrich (St. Louis, MO, USA) unless otherwise specified. Ca^2+^ and Mg^2+^ depleted PBS filtered using a 0.02 μm filter was used throughout the study.

### Human samples

One mL aliquots of archival serum (n = 6), plasma (n = 2), and urine (n = 3) from patients with genitourinary malignancies (bladder, kidney and prostate cancers), were obtained from the University of Cincinnati Biorepository. The study was carried out in accordance with the principles of the Declaration of Helsinki under an approved protocol of the institutional review board of University of Cincinnati (IRB# 2015-2364). Written informed consent was obtained and documented for all patients; samples were deidentified for patient confidentiality. Two of the urine samples were from the same patient. Basic demographic and clinical characteristics are provided for each patient in Table S3.

### Device fabrication and assembly

Detail of design criteria and fabrication procedure for the iDEP device has been previously reported by our group^59^. Briefly, the device consists of an array of glass micropipettes for particle trapping and embedded microelectrodes for applying electric field on a poly-methyl-methacrylate (PMMA) substrate. A laser-assisted puller P2000 (Sutter Instrument Company, Novato, CA, USA) was used for fabrication of micropipettes with 2 μm pore diameters from thick-walled borosilicate glass capillaries (BF-100-50-15, Sutter Instrument, Novato, CA, USA). The following parameters were used to obtain identical pores from all pulling events: Heat 350, Filament 4, Velocity 20, Delay 200, Pulling 0. Microelectrodes were fabricated using two photolithography processes followed by oxygen reactive ion etching (O_2_ RIE) and electroplating. The wafer was then diced into five strips of eight individual chips (MPE Inc., Greenville, TX, USA). A computer numerical control (CNC) 5100-S CNC milling machine (Microlution, IL, USA) was used to fabricate the PMMA substrate with alignment holders. Each electrode was soldered with copper wire for all chips.

### sEVs isolation using iDEP device

sEVs were simultaneously isolated from various biofluids, without any pre-purification procedures, for downstream analysis via flow cytometry and miRNA-seq using the iDEP device. Each device strip consists of eight chips and holds eight micropipettes for high throughput sEVs extraction, enabling extraction of 400 μL biofluid in ~ 20 min. Micropipettes were backfilled with 1 × PBS buffer using a 33-gauge Hamilton syringe needle and positioned on the PMMA substrate. The tip side and base side chamber of each micropipette was injected with 50 μL biofluid and 1 × PBS, respectively. sEVs from the biofluids were trapped at the tip by applying a 10 V/cm direct current (DC) for 10 min followed by a release in 25 μL 1 × PBS by reversing the applied voltage for another 10 min. 1 mL biofluids were simultaneously processed using 3 iDEP devices and parallel aliquots were performed to purify sEVs in 500 μL PBS. sEVs isolates were stored at − 80 °C for further analysis.

### Nanoparticle tracking analysis (NTA) and transmission electron microscopy (TEM)

Purified sEVs diluted in PBS at a dilution of 1:1000 from serum and plasma and 1:40 from urine samples were analyzed by nanoparticle tracking analysis (NTA) using a NanoSight NS300 instrument (Malvem, Worcestershire, UK) and the NTA 3.1 software. Camera level 14 and detection threshold 5 were used for instrument settings. Five 60-s recordings were obtained per sample. All post-acquisition functions were at default settings to output the mean, mode, standard deviation, and estimated concentration for each particle size.

sEVs were also imaged using a Talos L120C transmission electron microscope (TEM; Thermo Fisher Scientific, Waltham, MA, USA). Prior to sample loading, carbon-coated TEM grids were made hydrophilic with a plasma glow discharger unit (PELCO easiGlow; Ted Pella Inc., Redding, CA, USA). 3 μL of sEVs isolate was added to the grid for 60 s, and excess sample was removed by gently blotting the grid to a filter paper. The grid was exposed to 50 μL of 2% uranyl acetate (UA), followed by a wash in deionized water, with excess solution blotted between subsequent washes. The grid was subsequently incubated in 2% UA wash solution for 60 s prior to air drying. Excess solution was aspirated using a micro-vacuum pump. sEVs adsorbed onto the grid were visualized via TEM at 120 kV. Images were acquired using a 4K × 4K Ceta CMOS camera.

### Super resolution microscopy

Sub-diffraction limit resolution images of single sEVs were obtained using a Nanoimager S Mark II microscope (Oxford Nanoimaging, Oxford, UK) equipped with a 100 ×, 1.4 NA oil immersion objective, an XYZ closed-loop piezo 736 stage, and dual or triple emission channels split at 640 and 555 nm. 1 × 10^9^ sEVs in 1 mL were stained with 5 μg/mL tetramix of antibodies supplied in the EV profiler kit (Oxford Nanoimaging, Oxford, UK) including anti-CD63-Cy38 (800-00005, Oxford Nanoimaging), anti-CD81-AlexaFluor647 (800-00006, Oxford Nanoimaging), and anti-CD9-Atto488 (800-00007, Oxford Nanoimaging). Samples were processed according to manufacturer’s instructions to immobilize the stained sEVs on chips provided with the EV profiler kit. Three fields of view were recorded for each sample using direct stochastical reconstruction microscopy (dSTORM). Analysis was performed using algorithms including filtering, drift correction, and DBScan clustering developed by ONI via the Collaborative Discovery (CODI) platform.

### Conventional flow cytometry

sEVs analysis via flow cytometry was performed using an Aurora flow cytometer (Cytek, Fremont, CA, USA) at the Research Flow Cytometry Core at Cincinnati Children’s Hospital Medical Center (CCHMC). For sample preparation, 30 μL of sEVs isolate (~ 1 × 10^11^/mL) were aliquoted for unstained (control) and stained samples with each sample diluted to 200 μL using 1 × RNAse-free phosphate-buffered saline (PBS; ThermoFisher, Waltham, MA, USA). All samples were stained using the following antibodies: CD63-FITC (clone: H5C6, 353005, Biolegend, San Diego, CA, USA), CD81-APC (clone: 5A6, 349509, Biolegend, San Diego, CA, USA), and Isotype-IgG1 FITC (MOPC21, 400109, Biolegend, San Diego, CA, USA). Controls were prepared by adding 0.4 μg/mL antibody to PBS to confirm no interference of free-floating antibodies with data acquisition. The initial acquisition gate was established using PS-COOH beads of varying sizes (40, 80, 100, 500, and 800 nm; Phosphorex Inc., Hopkinton, MA, USA) and by adjusting the photo multimeter tube (PMT) voltage for forward-scatter (FSC-H) and side-scatter (SSC-H) to obtain optimal resolution for different sizes. Data were collected for 100 μL/sample for all samples. The flow rate was set to low and threshold count set at 5000. Data analysis was performed using the FlowJo software (FlowJo LLC, Ashland, OR, USA).

### ImageStream^x^

Advanced imaging flow cytometry analysis was performed for sEVs isolates using an ImageStream^x^ Mark II imaging flow cytometer (Amnis, Seattle, WA, USA) at the CCHMC Research Flow Cytometry Core. Twenty microlitre of each sEVs isolate (~ 1 × 10^11^/mL) was diluted to a total volume of 35 μL using 1 × PBS and aliquoted for unstained (control) and stained samples. sEVs were stained with CD63-FITC (clone: H5C6, 353005, Biolegend, San Diego, CA, USA), CD81-APC (clone: 5A6, 349509, Biolegend, San Diego, CA, USA), and Isotype-IgG1 FITC (MOPC21, 400109, Biolegend, San Diego, CA, USA) in the dark for 1 h. Samples were stained with 0.3 μg/mL antibody. Negative controls for antibody-only, unstained and Isotype samples and positive controls for commercially available sEVs harvested from hTERT-immortalized mesenchymal stem cells and A549 NSCLC cell lines purchased from ATCC Inc. were acquired (Manassas, VA, USA). Lysis controls were prepared by incubating filtered PBS-diluted sEVs in 0.1% Triton X-100 (Thermo Scientific) for 15 min at room temperature and stained with antibodies in order to confirm the requirement for vesicles to be intact for labeling to occur. Data acquisition was performed with low-speed fluidics, high sensitivity, core size 7 μm, and magnification at 60 ×. PS-COOH beads (FITC) of varying sizes (40, 100, and 200 nm) were used as references to determine the initial data acquisition gate for sEVs. Channels Ch01 and Ch09 were used for brightfield (BF) data and Ch12 was used to collect side scatter data. Data were collected for 3 min/sample for all samples. IDEAS software (Amnis Corporation) was used for data analysis.

### RNA isolation and detection

The miRNeasy Micro kit (Qiagen, Valencia, CA) was used to extract total RNA from the sEVs isolates using the as per the manufacturer’s suggested protocol. The Agilent 6000 Pico Kit using Bioanalyzer was used to measure the size distribution and estimated RNA concentration.

### microRNA-sequencing

The miRNA library preparation and sequencing was performed by the Genomics, Epigenomics and Sequencing Core at the University of Cincinnati College of Medicine. The NEBNext Multiplex Small RNA Library Prep kit (New England BioLabs, Ipswich, MA) was used for library preparation as per the manufacturer’s protocol with modifications to increase small RNA detection sensitivity and specificity for precise library size selection. Indexing and library enrichment was performed by 15 cycles of PCR and 10 μL PCR mix was pulled from each library, followed by column clean up. Pooled samples were mixed with 135 bp and 146 bp custom-designed DNA ladder corresponding to miRNA library with 16–27 nt. Precise size selection of 135–146 library was performed using agarose gel electrophoresis and quantified by NEB Next Library Quant kit (NEB) using Quant Studio 5 real-time PCR system (Thermo Fisher, Waltham, MA). A few million reads using a NextSeq550 sequencer (Illumina, San Diego, CA) as an initial round of sequencing to quantify the relative concentration of each library. The volume of each library was adjusted to generate equal reads from each sample for the final data analysis. Demultiplexed reads were pre-processed and trimmed to remove adapters fastp^60^. FASTQ files were processed according to ENCODE recommendations and aligned to human miRNA sequences (GRCh38/hg38) using the STAR aligner^61^. DESeq2 was utilized for normalization due to nucleotide lengths^62^.

### Supplementary Information

Supplementary Information.

## Data Availability

All miRNA sequencing datasets generated and/or analyzed during the current study are available in the ArrayExpress repository under accession code E-MTAB-13288.
