# Supplementary material for: Rapid purification and multiparametric characterization of circulating small extracellular vesicles utilizing a label-free lab-on-a-chip device
Source: Sci Rep. 2023 Oct 25;13:18293. doi: 10.1038/s41598-023-45409-4 (PMC10600140; doi:10.1038/s41598-023-45409-4)
Supplement: Supplementary file 1 — Supplementary Information. [file 41598_2023_45409_MOESM1_ESM.docx]

**Rapid purification and multiparametric characterization of circulating small extracellular vesicles utilizing a label-free lab-on-a-chip device**

Authors

Manju Sharma^1,+^, Maulee Sheth^1,+^, Holly M. Poling^1,2^, Damaris Kuhnell^3^, Scott M. Langevin^3,4^, Leyla Esfandiari^1,3,4,5*^

Affiliations:

*^1^Department of Biomedical Engineering, College of Engineering and Applied Sciences, University of Cincinnati, Cincinnati, OH, USA*

*^2^Division of Pediatric General and Thoracic Surgery, Cincinnati Children’s Hospital Medical Center, Cincinnati, OH, USA*

*^3^Department of Environmental & Public Health Sciences, College of Medicine, University of Cincinnati, Cincinnati, OH, USA*

*^4^University of Cincinnati Cancer Center, Cincinnati, OH, USA*

*^5^Department of Electrical Engineering and Computer Science, College of Engineering and Applied Sciences, University of Cincinnati, OH, USA*

**^+^ Authors contributed equally to work**

**^*^ Corresponding author**

**A. Sample-wise nanoparticle tracking analysis and RNA quality control results**

Table S1. Sample specific details including extraction conditions used, nanoparticle tracking analysis (NTA) results and electropherogram results indicative of exosomal RNA.

| `  Sample No. | Biofluid | Extraction conditions | | NTA results | | | Electropherogram for exosomal RNA |
| --- | --- | --- | --- | --- | --- | --- | --- |
|  |  | Sample  volume | Isolation  technique | Mean  (nm) | SD  (nm) | Concentration (#/mL) | RNA concentration (ng/mL) |
| 1 | Serum | 1 mL | iDEP | 114.9 | 54.6 | 1.16E+11 | 117 |
| 2 | Serum | 1 mL | iDEP | 76.0 | 20.2 | 6.20E+10 | 43 |
| 3 | Urine | 1 mL | iDEP | 189.3 | 97.7 | 7.73E+11 | 82 |
| 4 | Serum | 1 mL | iDEP | 61.2 | 18.2 | 5.46E+11 | 59 |
| 5 | Serum | 1 mL | iDEP | 100.9 | 18.2 | 1.18E+11 | 87 |
| 6 | Serum | 1 mL | iDEP | 99.4 | 52.4 | 1.26E+11 | 133 |
| 7 | Urine | 1 mL | iDEP | 133.0 | 50.4 | 1.18E+10 | 113 |
| 8 | Urine | 1 mL | iDEP | 97.9 | 37.0 | 3.45E+09 | 119 |
| 9 | Serum | 1 mL | iDEP | 78.5 | 23.6 | 5.17E+11 | 132 |
| 10 | Plasma | 1 mL | iDEP | 92.4 | 49.4 | 9.55E+10 | 122 |
| 11 | Plasma | 1 mL | iDEP | 110.0 | 58.0 | 1.09E+12 | 76 |

**B. Nanoparticle tracking analysis results**

Table S2. Representative biofluid-specific NTA results including graph, mean, standard deviation and particle concentration.

| **NTA graph** | **Statistics** |  |
| --- | --- | --- |
| 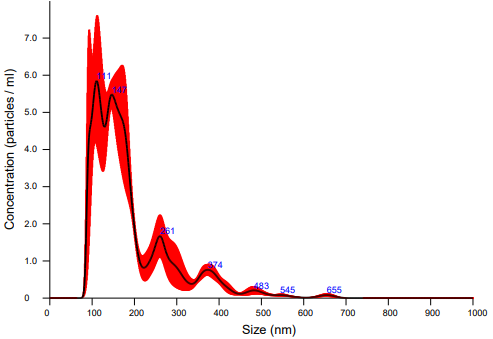 | Sample No.: | 3 (urine) |
|  | Mean (nm): | 189.3 |
|  | SD (nm): | 97.7 |
|  | Concentration (#/mL): | 7.73E+11 |
| 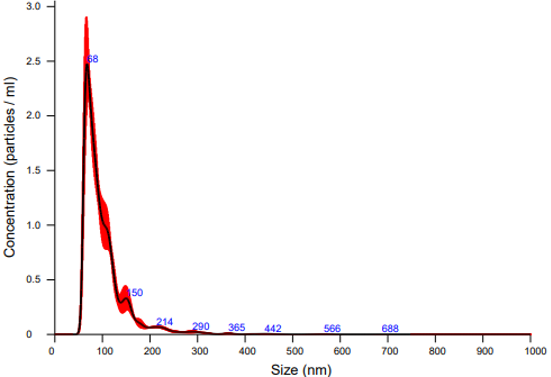 | Sample No.: | 5 (serum) |
|  | Mean (nm): | 100.9 |
|  | SD (nm): | 18.2 |
|  | Concentration (#/mL): | 1.18E+11 |
| 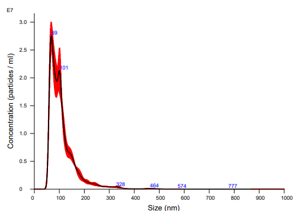 | Sample No.: | 11 (plasma) |
|  | Mean (nm): | 110.0 |
|  | SD (nm): | 58.0 |
|  | Concentration (#/mL): | 1.09E+12 |

**C. Patient details**

Table S3. Patient specific details including age, race, sex, histology, type of biofluid, cancer site, pathology stage and grade. Summary and Clinical stage information were not available for samples.

| Sample No. | Biofluid | Age | Race | Sex | Cancer site | Histology | Pathology Stage | Pathology Grade or Gleason Score (prostate) |
| --- | --- | --- | --- | --- | --- | --- | --- | --- |
| 1 | Serum | 63 | White or Caucasian | Male | Kidney | Clear cell renal cell carcinoma | pT1a NX MX | G2 (Fuhrman Nuclear Grade) |
| 2 | Serum | 63 | White or Caucasian | Female | Bladder | Transitional cell carcinoma | pT3b N0 MX | High Grade |
| 3 | Urine | 61 | Black or African American | Male | Prostate | Adenocarcinoma | pT3b N0 MX | Gleason score 3+4=7; Grade Group 2 |
| 4 | Serum | 43 | Black or African American | Female | Kidney | Clear cell papillary renal cell carcinoma | pT1a NX | G1 (WHO/ISUP Grade) |
| 5 | Serum | 56 | White or Caucasian | Male | Prostate | Adenocarcinoma | pT3b N0 | Gleason Score 4+5=9; Grade Group 5 |
| 6 | Serum | 62 | White or Caucasian | Female | Bladder | Carcinoma, NOS | ypTa N0 | High Grade |
| 7 | Urine | 63 | White or Caucasian | Male | Prostate | Adenocarcinoma | pT3b N1 MX | Gleason score 3+4=7; Grade Group 2 |
| 8 | Urine | 63 | White or Caucasian | Male | Prostate | Adenocarcinoma | pT3b N1 MX | Gleason score 3+4=7; Grade Group 2 |
| 9 | Serum | 61 | Black or African American | Male | Prostate | Adenocarcinoma | pT3b N0 MX | Gleason score 3+4=7; Grade Group 2 |
| 10 | Plasma | 63 | White or Caucasian | Male | Prostate | Adenocarcinoma | pT3b N1 MX | Gleason score 3+4=7; Grade Group 2 |
| 11 | Plasma | 63 | White or Caucasian | Male | Kidney | Clear cell renal cell carcinoma | pT1a NX MX | G2 (Fuhrman Nuclear Grade) |

**D. Exosomal RNA quality control**

Table S4. RNA quality including electropherograms and RNA concentration for each type of biofluid. RNA quality graphs represent florescence on y-axis and RNA size on x-axis.

| **RNA Quality** | **Electropherogram** | **Statistics** | |
| --- | --- | --- | --- |
| 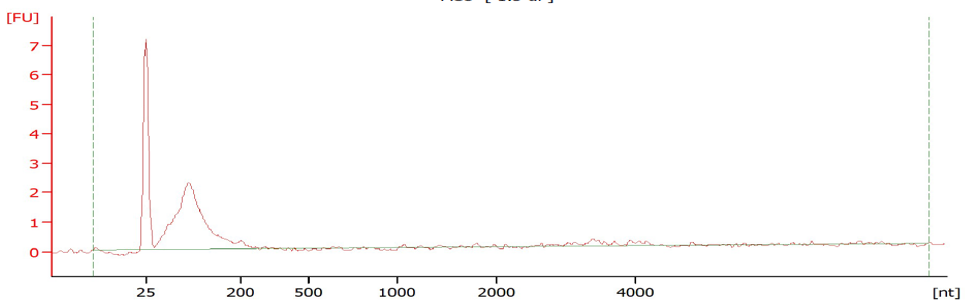 | 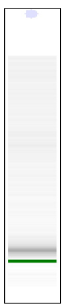 | Sample No.:  RNA conc.: | 1 (serum)  117 ng/mL |
| 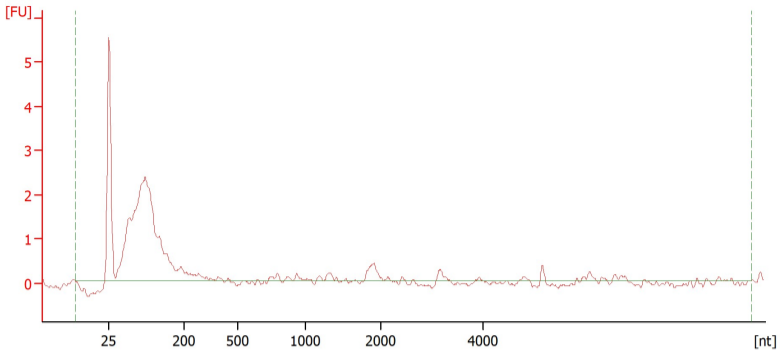 | 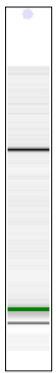 | Sample No.:  RNA conc.: | 7 (urine)  113 ng/mL |
| 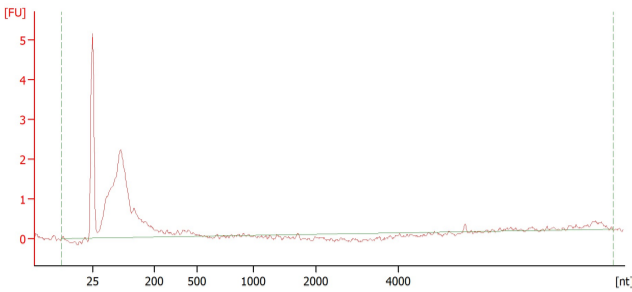 | 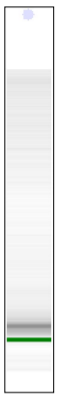 | Sample No.:  RNA conc.: | 10 (plasma)  122 ng/mL |

**E. Sample of Transmission Electron Microscopy (TEM) of extracted sEVs from serum**

Figure S1. Representative transmission electron microscopy (TEM) of serum sEVs (Sample 1) isolated using the iDEP device

**F. Sample of 3-color dSTORM images of extracted sEVs from serum**


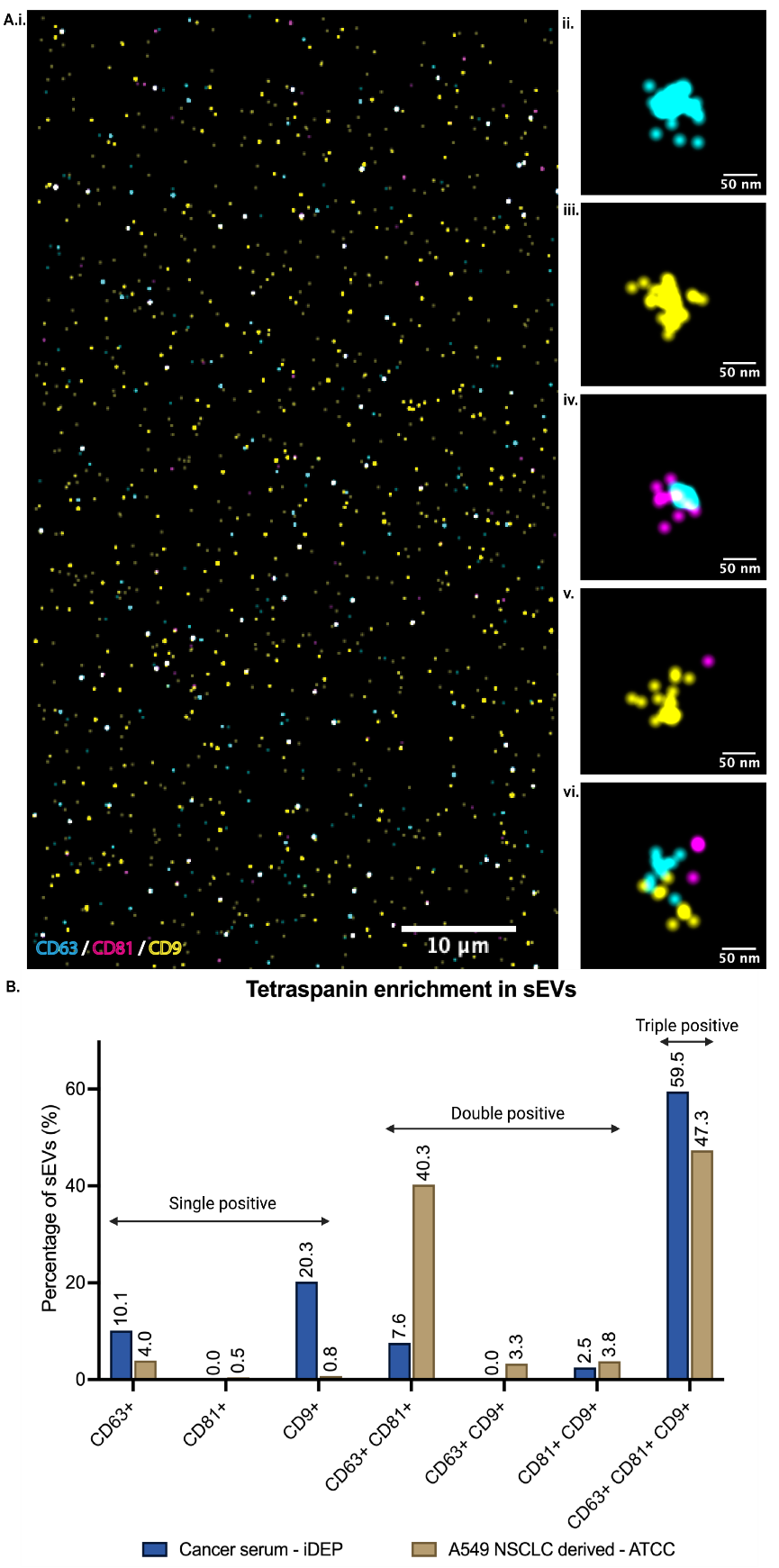


**Figure S2.** **A)** 3-color dSTORM images of CD63-568, CD81-647, and CD9-488 EVs isolated using the iDEP device from serum of cancer patient. i. Magnification showing a larger view of single EVs going down to 10-micron scale bar, ii-v. Single EV at 50 nm scale bar presenting only one tetraspanin surface marker (ii-iii), two markers (iv-v), and three markers (vi). **B)** Graph showing the percentage of EVs with single, double, and triple markers. A549 NSCLC derived sEVs from ATCC Inc. are represented as positive control.

**G. Imagestream^x^ detergent lysed antibody-stained controls for purified sEVs**

**
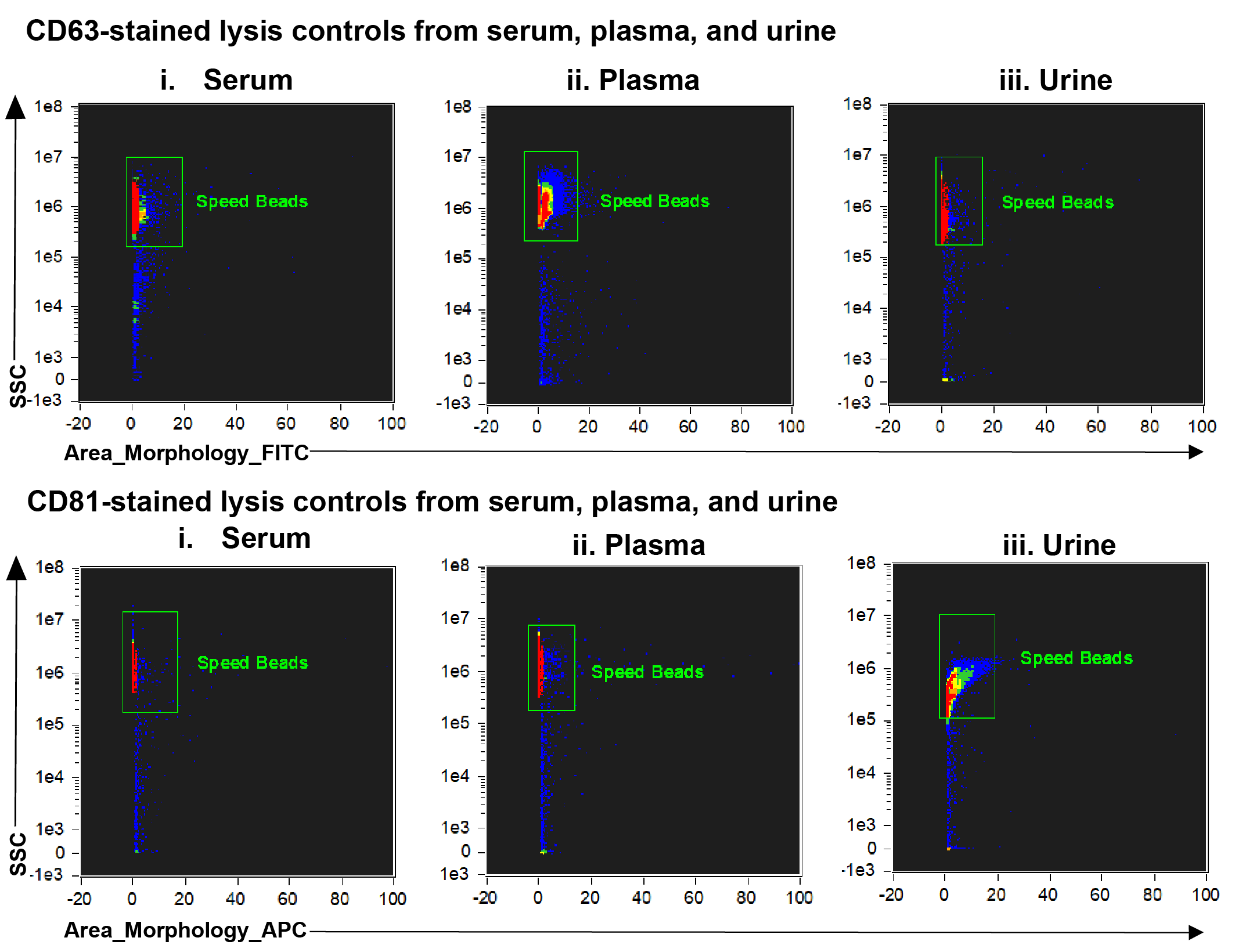
**

Figure S3. Lysed CD63- and CD81-stained sEVs from serum, plasma, and urine samples.

**H. Clustering column dendrogram from miRNA sequencing analysis**

**
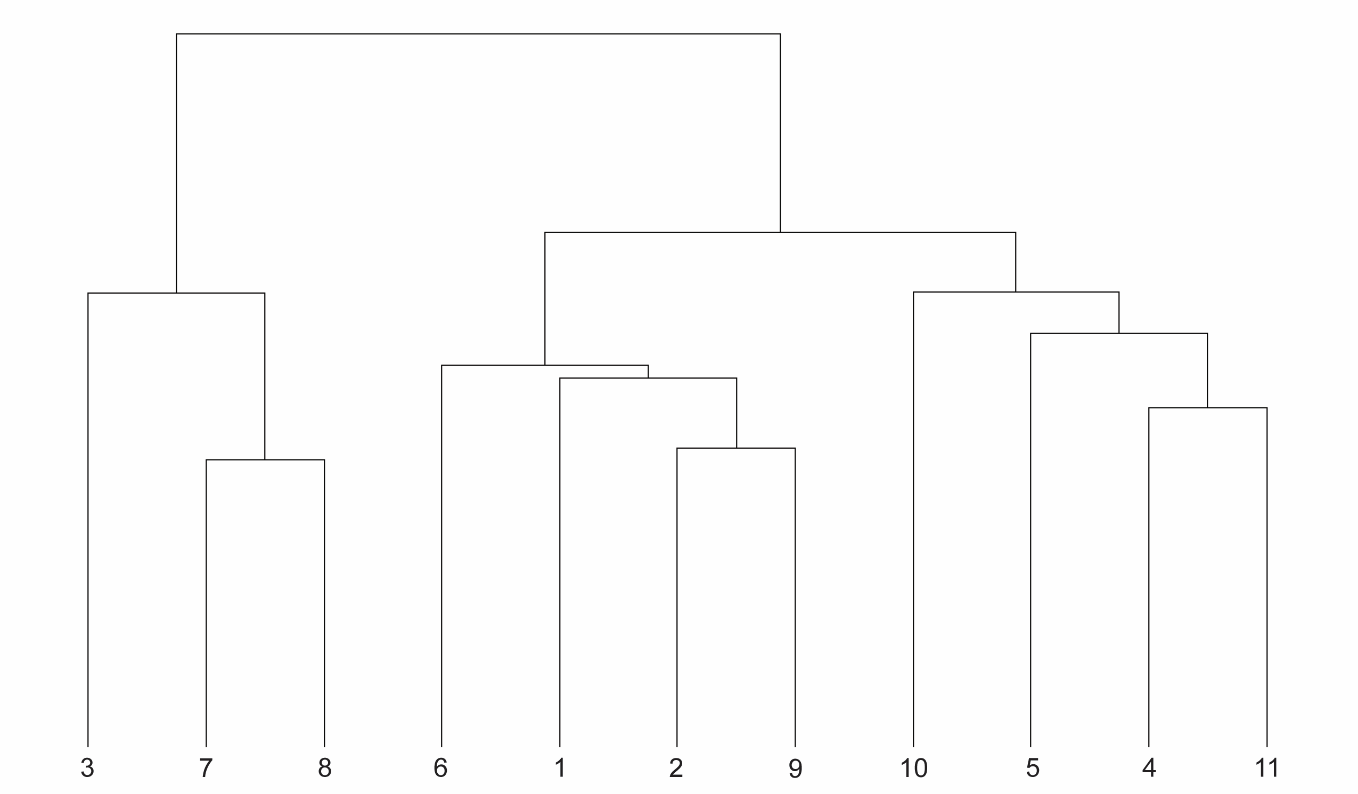
**

Figure S4. Hierarchical clustering of miRNA sample profiles from serum, plasma, and urine.
